# Regulation of red fluorescent light emission in a cryptic marine fish

**DOI:** 10.1186/1742-9994-11-1

**Published:** 2014-01-08

**Authors:** Matthias F Wucherer, Nico K Michiels

**Affiliations:** 1 Animal Evolutionary Ecology, Department of Biology, Faculty of Science, University of Tübingen, Auf der Morgenstelle 28, D-72076, Tübingen, Germany

**Keywords:** Pigmentation, Fluorescence, Tripterygiidae, Triplefin blenny, Camouflage, Signalling, Marine fishes

## Abstract

**Introduction:**

Animal colouration is a trade-off between being seen by intended, intra- or inter-specific receivers while not being seen by the unintended. Many fishes solve this problem by adaptive colouration. Here, we investigate whether this also holds for fluorescent pigments. In those aquatic environments in which the ambient light is dominated by bluish light, red fluorescence can generate high-contrast signals. The marine, cryptic fish *Tripterygion delaisi* inhabits such environments and has a bright red-fluorescent iris that can be rapidly up- and down-regulated. Here, we described the physiological and cellular mechanism of this phenomenon using a neurostimulation treatment with KCl and histology.

**Results:**

KCl-treatment revealed that eye fluorescence regulation is achieved through dispersal and aggregation of black-pigmented melanosomes within melanophores. Histology showed that globular, fluorescent iridophores on the anterior side of the iris are grouped and each group is encased by finger-like extensions of a single posterior melanophore. Together they form a so-called *chromatophore unit*. By dispersal and aggregation of melanosomes into and out of the peripheral membranous extensions of the melanophore, the fluorescent iridophores are covered or revealed on the anterior (outside) of the iris.

**Conclusion:**

*T. delaisi* possesses a well-developed mechanism to control the fluorescent emission from its eyes, which may be advantageous given its cryptic lifestyle. This is the first time chromatophore units are found to control fluorescent emission in marine teleost fishes. We expect other fluorescent fish species to use similar mechanisms in the iris or elsewhere in the body. In contrast to a previously described mechanism based on dendritic fluorescent chromatophores, chromatophore units control fluorescent emission through the cooperation between two chromatophore types: an emitting and an occluding type. The discovery of a second mechanism for fluorescence modulation strengthens our view that fluorescence is a relevant and adaptive component of fish colouration.

## Introduction

Colour vision is widespread in the animal kingdom and has driven the evolution of body colouration [1]. Conspicuous colouration is often associated with mate choice [2-4], warning signals [5-7] or species recognition and communication [8-11]. Light emission through bioluminescence has also been proposed as a detection and communication mechanism [12]. Cryptic colouration patterns, however, are required to hide from visual predators or to approach potential prey [9,10,13,14]. Many colour patterns are therefore a compromise between being visible to the intended receivers while avoiding the attention of the unintended, e.g. predators [15-18]. These opposing forces drive the evolution of adaptive, context-dependent body colouration [19-21]. Cephalopods, bony fishes, amphibians and reptiles achieve this by complex arrangements of pigmented skin cells or chromatophores, such as melanophores (black), xanthophores (yellow), erythrophores (red) and iridophores (structural colours) [9,21,22]. It is the complex interplay of these cell types that generates colour appearances ranging from well-camouflaged lizards to the most brightly coloured reef fishes [23].

Colourful aquatic animals face the constraint that water absorbs long wavelengths (> 600 nm) to a much higher degree than short and medium wavelengths [24]. Consequently, many marine environments below 10–20 m are essentially blue-green. In the absence of long wavelengths, however, none of the usual reflective pigmentation mechanisms can generate shades of red (600–700 nm). Fluorescent pigments can restore those “lost” colours by absorbing the available light and re-emitting it at a longer wavelength [24-26]. This process is different from bioluminescence, where photons are generated through a chemical reaction. In many fishes and cephalopods, the latter function is provided by microbial symbionts [26-28].

Many marine animal phyla are known to fluoresce, with cnidarians being the best investigated group [29-34]. We recently showed that a number of fish taxa show fluorescence in the near red range (600–630 nm) and proposed fluorescent pigmentation as a new signalling mechanism or a mechanism for highlighting prey or predators with reflecting eyes [25]. Knowing that many marine fishes are trichromatic [35,36], red fluorescence has the potential to generate strong, visible chromatic contrasts at depth. Conspicuous signals, however, may attract attentive predators that can see them. We would therefore expect that fluorescence can be modulated on demand – as is the case for (eye) colouration in other marine fishes [37]. Recently, we described a first pigment cell type with this feature: a dendritic, red fluorescent chromatophore in the clear fins of the pygmy goby *Eviota atriventris* (previously misidentified as *Eviota pellucida,* see [38]) that actively disperses and aggregates intracellular fluorescent particles, modulating the brightness of the fluorescent emissions [39].

**Figure 1:**
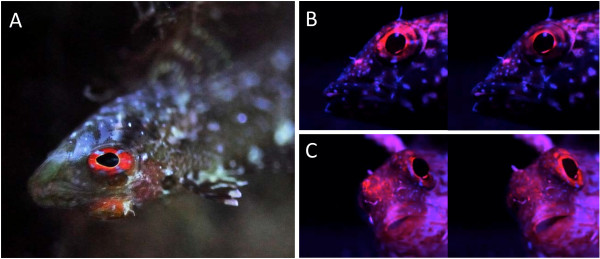
**Red fluorescence in the iris of *Trypterygion delaisi*.** Illustration of fluorescence in *Tipterygion delaisi***A**. Individual showing red fluorescent eye-ring (= iris) under natural conditions (at -23 m, upside down under shady overhang, STARESO, Corsica, using a Nikon D700 DSLR, image taken without flash or filter and manually adjusted white balance). **B** and **C** are two pairs of images, each of which was taken with a 5 *s* delay showing rapid changes in the brightness of iris fluorescence in the laboratory using blue illumination and a red filter for viewing.

**Figure 2:**
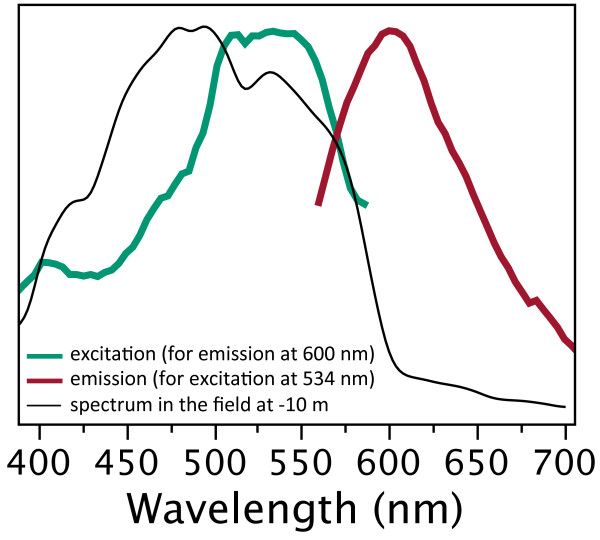
**Maximum-normalised excitation and emission spectra in *T. delaisi *(Y-axis in arbitrary units, ranging from minimum to maximum).** Fluorescence peaks at 600 nm (red line). Excitation is most efficient between 500 and 570 nm (green line). The excitation spectrum overlaps strongly with the ambient light spectrum (taken at noon on a sunny day in July at STARESO, Calvi, Corsica, in -10 m depth (black, thin line). Excitation and emission were measured using a spectrofluorometer (QuantaMaster QM-40, Photomed, Germany).

Here, we describe a second mechanism for fluorescence modulation in the cryptic Mediterranean triplefin, *Tripterygion delaisi xanthosoma* (Tripterygiidae), a species that can be found predominantly at depths between -6 and -40 m. It generally prefers shady rock faces, crevices and overhangs, particularly in the upper part of its depth distribution [40,41]. At these sites, the long wavelength component of sunlight has been largely absorbed by the water column, and scatter from the open water dominates the ambient light [24,42]. The eyes of *T. delaisi* have a strongly red fluorescent iris, similar to that of several other small, benthic fishes [25] (Figures 1A and 2). It is clearly visible to a SCUBA diver in the field – without the aid of filters. Interestingly, *T. delaisi* has the ability to rapidly vary eye fluorescence brightness in the laboratory (Figure 1B, C), something we have also observed in the field. We suspected that this mechanism involves melanophores, which are known to occlude reflective pigment cells in the skin of many other species [43-47]. We tested this by triggering the aggregation of melanosomes in melanophores using potassium ions K + [21,22,48] and investigating their interrelationship with the fluorescent cells in the iris. We also characterise the cell complex that generates and controls fluorescence by means of histology and 3D-reconstruction.

## Results

### Change in fluorescence brightness after KCl treatment

**Figure 3:**
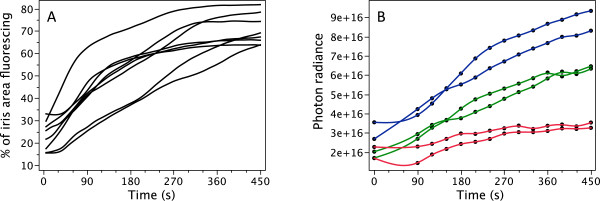
**Change in fluorescence intensity after application of KCl. A**. Change in percentage of fluorescing iris area over a 450 s period (one measurement per 5 s) averaged for 25 *s* time intervals for 8 eyes from 8 different individuals. KCl was added at time point 0 *s*. **B**. Change in iris fluorescence brightness over time in 6 eyes from 3 individuals expressed as total photon radiance (photons.s^-1^.sr^-1^.m^-2^) in the emission range 550–700 nm at each time point. Colours indicate individuals.

**Figure 4:**
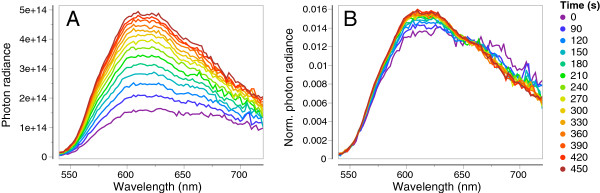
**Iris fluorescence brightness expressed as photon radiance, photons.s**^**-1**^**.sr**^**-1**^**.m**^**-2**^**.nm**^**-1**^**) plotted as a function of wavelength in time steps (see colour legend) starting with the application of KCl at time = 0 s.** Data were averaged for 6 eyes from 3 individuals. **A**. Absolute spectra. **B**. Area-normalized spectra (each value of a spectrum divided by the sum of all values of that spectrum).

After K^+^ treatment, the fluorescent area increased significantly from 23.3 ± 6.7% (mean ± SD) of the iris surface in neutral saline to 71.5 ± 6.8% after KCl-treatment (3A, comparison of start and end values using paired t-test *t* = 14.1, *df* = 7, *P* < 0.0001). This was confirmed by total photon radiance measurements of eye fluorescence, which increased significantly from on average 1.6 × 10^16^ at 0 s to 3.8 × 10^16^ photons s^-1^ sr^-1^ m^-2^ at 450 s (Figure 3B, paired t-test *t* = 4.79, *df* = 5, *P* = 0.0049, eyes grouped per individual). Figure 4 shows that the change in brightness due to the K^+^ treatment affected the whole emission spectrum equally. When normalising all spectra, no obvious spectral shifts can be distinguished (Figure 4B).

### Iris histology

**Figure 5:**
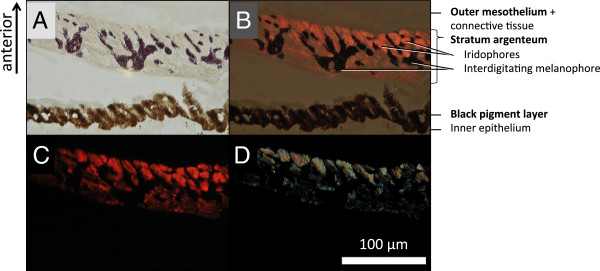
**Change in fluorescence spectrum after application of KCl.** Micrographs of a section through the iris under bright field **(A)**, bright field and fluorescence **(B)**, fluorescence **(C)** and polarized light **(D)**, showing that the fluorescence emanates from the *stratum argenteum*, a layer of iridophores with guanine crystals (see **D** compared to **B** and **C**) (labelling in accordance with [37], p. 47). This layer is invaded by finger-like extensions of melanophores at the posterior side of the *stratum argenteum* (dark). The anterior (outward facing) side is at the top.

**Figure 6:**
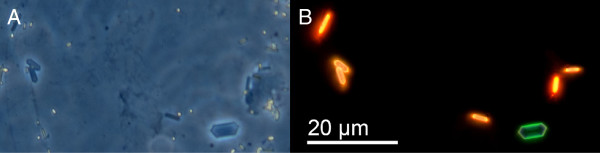
**Guanine crystal platelets from the iris of *T. delaisi *under light microscopy. A**: phase contrast. **B**: fluorescence. Note the weak blue-green fluorescence of the regular guanine crystal at the bottom right, which lacks red fluorescent pigment present in the others. Shapes vary a lot in these crystals, but do not seem to differ systematically between fluorescent or non-fluorescent forms.

Histological sections of the iris showed that fluorescence is located in the *stratum argenteum*, anterior to a densely packed black-pigmented melanophore layer. As in many other teleosts, the *stratum argenteum* mainly consists of densely packed, globular iridophores ([49], Figure 5). Iridophores are chromatophores that lack regular pigments, but contain stacks of thin, crystalline platelets made of a mixture of guanine and hypoxanthine [22]. In other fishes these are known to generate silvery iridescence [50] or structural colouration through multilayer thin-film interference [22,51,52]. In the iris of *T. delaisi*, we identified the platelets as the structures containing the fluorescent pigment (Figure 6).

**Figure 7:**
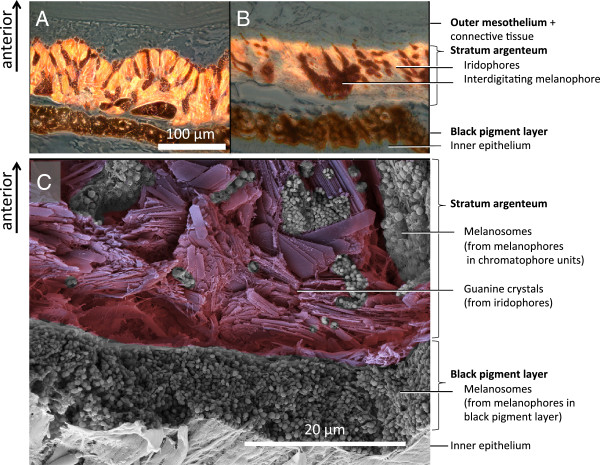
**Sections of *T. delaisi *irides, showing chromatophore location.** Micrographs of the iris with melanophores before **(A)** and after **(B)** KCl treatment. Before KCl treatment, melanosome projections extend over the top of the fluorescent structures and suppress fluorescent emission. After KCl treatment **(B)** they are retracted and facilitate fluorescent emission. **C**: SEM picture of a fractured, untreated iris with similar layering, showing the contents of the chromatophores: Guanine crystal platelets from iridophores, and melanosomes (black pigment vesicles) from melanophores.

**Figure 8:**
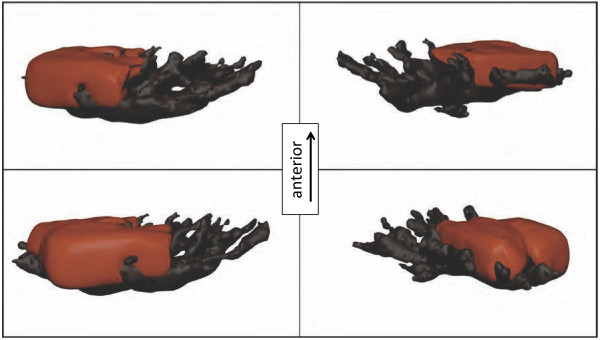
**3D-Model of a reconstructed fluorescent chromatophore unit with one melanophore (black) embracing four fluorescent globular iridophores (red, two shown, two others omitted).** Sample taken at an intermediate state with not yet fully aggregated melanosomes.

Independent of the posterior, dense melanophore layer, the *stratum argenteum* also possesses a number of melanophores on its posterior side. These melanophores show finger-like processes, which interdigitate and cover the anterior side of the fluorescent iridophores (Figures 5, 7 and 8). In samples taken before K^+^ treatment (Figure 7A), more melanosomes were located in the anterior, dendritic processes, darkening the outside of the iris. In this situation, the posterior somata of the melanophores were smaller. In the post-treatment state, almost all melanosomes were withdrawn from the outside of the iris, accumulating in the posterior body of the melanophores (Figure 7B). These observations indicate that in the pre-treatment, “dark” state, aggregation of melanosomes on the outer surface of the iris prevents ambient incoming light from reaching the iridophores. In addition, melanosomes in the finger-like extensions presumably block fluorescence excitation as well as emission inside the *stratum argenteum*. In the aggregated state, ambient light can reach and be absorbed by the fluorescent iridophores and then re-emitted as red light. Modulation of fluorescence does not require a change of the reflective configuration of the iridophores, but can be achieved by occlusion by melanosomes only. A single melanophore can be associated with multiple iridophores and a single iridophore can be covered by extensions coming from different melanophores. Figure 7C shows a cross-section of the untreated iris under SEM, confirming that the iridophore layer is fully packed with guanine crystals, interdigitated with melanosomes-filled extensions from melanophores situated at the posterior side of the *stratum argenteum*. Figure 8 shows a 3D-reconstruction based on sections such as those shown in Figures 7A-B.

## Discussion

The long wavelength fluorescence in the iris of *T. delaisi* is emitted by red fluorescent guanine crystals, which are stacked in iridophores, the dominant cell type in the *stratum argenteum.* The brightness of fluorescence is modulated by rapid (2–6 min) transport of melanosomes between the soma of posterior melanophores and their finger-like extensions that stretch anteriorly through the *stratum argenteum* and cover the iridophores anteriorly. Given that the whole process can be controlled by the presence or absence of K^+^ ions indicates that it is under neuronal control [48]. Histological investigation revealed that iridophores and melanophores cooperate in so-called *chromatophore complexes* or *units*, whereby multiple cells of one type interact and overlap with multiple cells of the other kind. This is mainly due to the network-like structure generated by the cellular extensions of the melanophores. Such complexes are known to control colouration in the skin of fishes, amphibians and reptiles [21,23,53]. Chromatophore units often consist of a xanthophore and/or iridophore plus a melanophore [54]. In *T. delaisi* only two kinds of chromatophores are involved. In all cases, posterior melanophores aggregate or disperse melanosomes, thereby controlling the brightness [55] of the associated anterior iridophores. Our study now shows that such units are also used to control fluorescent emission.

The presence of red fluorescence in guanine crystals is a feature known from other red fluorescent fish species, including pipefish of the genus *Corythoichthys*[25]. The iris of *Corythoichthys paxtoni* has a structure similar to that of *T. delaisi*[56]. It is therefore quite likely that some pipefish also possess chromatophore units similar to the ones described here. Own field observations indicate that other triplefin species of the genera *Tripterygion*, *Helcogramma* and *Enneapterygius* and gobies belonging to the genus *Ctenogobiops* and *Istigobius* also show red fluorescent irides that can be quickly darkened or lightened (pers. observations, N. K. Michiels), suggesting that this mechanism is not limited to a few rare cases.

### An active role for iridophores?

Our data do not allow us to make conclusive inferences whether the observed changes in fluorescent emission are in part caused by conformational changes in the orientation or distance of the guanine platelets in the *stratum argenteum*. Motile iridophores are known from cephalopods [9] as well as from fishes with prominent iridescent or structural colour patterns [57-61]. In some of these cases, rapid (within seconds) changes in hue and brightness can be observed through conformational changes in motile iridophores. Although our data do not allow us to exclude that such a process may have contributed to the observed changes in brightness, the emitted hue remained unaffected under the conditions tested (Figure 4).

### Comparison with colour change mechanisms in cephalopods

An analogous, well-understood mechanism to modulate colour is the chromatophore organ in cephalopods [62]. It also consists of different cell types that cooperate to control colour [63]. In cephalopods, however, this is achieved by the action of radial muscles that change the shape of coloured chromatophores. Upon contraction, the muscles stretch and thereby display the pigmented content of a pigment sac inside the chromatophore [23,64]. Muscle relaxation leads to spontaneous contraction of the pigment sac, hiding the pigment [63,64]. Light passing through the system is reflected by iridophores that sit behind the complex.

### Possible function

As K^+^ stimulates action potentials in neurons, it is safe to conclude that *T. delaisi* modulates its fluorescence through a neuronally-controlled mechanism [65]. We observed that specimens of *T. delaisi* become darker when stressed or when inactive, but during spontaneous activity, particularly when foraging, the brightness of the iridial fluorescence visibly increases (unpublished observations). The brightness of fluorescence therefore likely constitutes a relevant cue that is possibly used in a foraging context. Since peak emission is in the near-red spectral range (590–610 nm), it is very likely that other fish can perceive the fluorescence of *T. delaisi*. Work in progress confirms that *T. delaisi* is a trichromat including a LWS receptor, making it likely that it can see its own fluorescence.

## Conclusion

Red fluorescence in the iris of *T. delaisi* is controlled by so-called chromatophore units, which consist of a melanophore and several fluorescent iridophores. Modulation of fluorescence is achieved by nervous control of the aggregation state of melanosomes in melanophores. The presence of this sophisticated control mechanism suggests that *T. delaisi* controls its fluorescence actively. This reduces the constraints raised by the colouration trade-off between contrast and camouflage at depths below -10 m. In shallower water, the same process will modulate the overall reflective properties of the iris.

## Material and methods

### *Fluorescence in* Tripterygion delaisi

*Tripterygion delaisi* (Tripterygiidae) occurs throughout the East-Atlantic Ocean and West-Mediterranean Sea. It is a small (up to 6 cm), cryptic, benthic predator feeding on micro-crustaceans between -5 and -40 m depth. When approached, individuals freeze rather than flee, indicating that they rely on their camouflage. Yet, *T. delaisi* has prominent red fluorescent irides (Figure 1). Its fluorescence is characterized by light absorption across a broad range of the ambient spectrum (Figure 2) and a peak emission at 600 nm (bright red). The relationship between fluorescence and ambient light will be published in detail elsewhere (Michiels *et al.* in prep.). All juvenile (< 1 cm) and adult stages show fluorescent irides throughout the year. The brightness of fluorescence can vary strongly and quickly (Figure 1). Pilot studies in the field and in the lab showed that downregulation is associated with inactivity or stress (e.g. when handled). Upregulation is often seen in the context of foraging.

Specimens of *T. delaisi* were collected in Corsica, France at the STARESO Marine Science Station, under the general sampling permit of the station. In Tübingen, Germany, fish were kept in individual divisions of a large flow-through aquarium (20°C, salinity 34%, pH 8.2). For the experiments, fish were decapitated, dissected and the eyes were stored in physiological saline as described by Wucherer and Michiels [39]. All work was carried out and recorded in accordance to German animal protection legislation.

### Temporal change in fluorescence area and brightness

Eyes were immersed in saline by placing them on a perforated 8 mm ∅ plastic platform in a 35 mm ∅ petri dish. They were investigated under a Leica MZ 16 F fluorescence stereomicroscope (band-pass filter with 530–560 nm for excitation). Fluorescent emission was viewed through a second, non-overlapping bandpass-filter (590–650 nm) and documented using a Nikon D300 DSLR. For each treatment, a 450 s time-lapse recording was taken providing an image every 5 s. At the start (0 *s*) we replaced the saline with a high KCl-solution (in mM: NaCl 78, KCl 50, CaCl2 1.8, MgCl2 1.8, D(+)-Glucose 5.6, Tris–HCl 5.0; pH 7.2). Total ion concentration was identical to the saline solution. Eyes from eight fish were analysed, one of each fish exposed to the KCl-treatment, the other one to the control treatment.

In order to calculate the fluorescent area, images were imported as stacks into ImageJ (v. 10.2, J. Rasband, NIH, USA), set to 8-bit grey-scale and inverted. A threshold was set to distinguish between fluorescent and non-fluorescent areas (black in the original image). Then the fluorescent area of every image was determined by counting the proportion of fluorescent pixels relative to those of the whole iris. For statistical analysis, time and the proportion of fluorescent area were entered into JMP (v. 9, SAS, USA).

Fluorescence brightness (photon radiance) was measured using a SpectraScan PR670 spectroradiometer (Photoresearch, Chatsworth, CA, USA). Six eyes were dissected from three fish and pre-treated as described above. Each eye was illuminated at an angle of 45° with an LED light source with peak emission at 455 nm (LLS455, Sandhouse, Dunedin, FL, USA). Fluorescence was measured from above, perpendicular to the iris with the measuring point aimed to exactly cover the complete iris (0.5° measurement angle). Measurements were taken before and at 8 time points after application of the KCl solution: 0, 90, 120, 180, 240, 300, 360, 420, 450 *s*. Data were summarized as the cumulative sum of photons.s^-1^.sr^-1^.m^-2^ across the emission range (500–750 nm) (= fluorescence brightness).

### Histology

Following KCl-treatment, eyes were fixed and embedded as described for fin tissue by Wucherer and Michiels [39]. We cut 1.5 μm sections parallel to the axis of vision (axial plane) using a diamond knife and mounted the sections in a non-fluorescent medium (Vectashield, Vector). Digital micrographs of each section were stacked to generate a 3D-reconstruction of the chromatophore unit (chromatophores and surrounding cells) using TrakEM2 [66,67]. The 3D-model was smoothed and vertices were reduced using Blender (v. 2.63a).

Sub-cellular structures in an iris cross-section were visualised by scanning electron microscopy of the fracture zone of an untreated freeze-dried *T. delaisi* iris by Oliver Meckes and Nicole Ottawa from Eye of Science GbR (http://www.eyeofscience.com) (Figure 6C).

### Ethical approval

Fish were sacrificed under permit no. ZO 1/10 from 18 Oct 2010, prolonged on 26 Nov 2011 till 15 Dec 2012 to the corresponding author. The permit was issued by the Animal Protection Office at the Regierungspräsidium, Referat 35, Konrad-Adenauer-Straβe 20, 72072 Tübingen, Germany.

## Competing interests

The authors declare that they have no competing interests.

## Authors’ contributions

MFW planned and collected most of the data presented. He also analysed and summarized the data and wrote the first drafts of the manuscript. NKM was the supervisor of the work, contributed data for Figure 2 and prepared the statistical analysis, the graphs and the text for publication. Both authors read and approved the final manuscript.
